# Syphilis Infection During Pregnancy: The Possible Effect on the Course of Pregnancy and Fetal Outcomes—A Case Report and Literature Review

**DOI:** 10.3390/biomedicines13010169

**Published:** 2025-01-13

**Authors:** Dovile Kielaite, Saule Januskiene, Virginija Paliulyte

**Affiliations:** 1 Faculty of Medicine, Vilnius University, 03101 Vilnius, Lithuania; 2 Clinic of Obstetrics and Gynaecology, Faculty of Medicine, Institute of Clinical Medicine, Vilnius University, 03101 Vilnius, Lithuania; virginija.paliulyte@mf.vu.lt

**Keywords:** case report, congenital infection, infection during pregnancy, pregnancy, sexually transmitted disease, syphilis

## Abstract

**Background/Objectives:** A wide range of syphilis-related pregnancy complications are encountered in clinical practice. Active surveillance of the epidemiological situation in different countries and a series of retrospective data analyses allow for a comprehensive assessment of the feasible consequences of syphilis infection during pregnancy. The negative effects of infection on reproductive health are also described. Risk-increasing factors (inadequate or late treatment, partner coinfection) and protective factors (timely diagnostics and treatment) are distinguished. The importance of adequate and timely management as well as the accessibility of healthcare and socioeconomic status, which influence health outcomes, are stressed. This article presents a rare case of untreated syphilis infection during pregnancy. The infection was diagnosed during the first antenatal visit; how-ever, treatment was not initiated. At the 33rd week of gestation, the patient was admitted to the hospital because of sparse bloody vaginal discharge. Following sudden fetal hypoxia, an urgent cesarean section was performed at 33 weeks of gestation. A preterm newborn was delivered in critical condition, and congenital syphilis was diagnosed. **Methods:** We searched the PubMed, Cochrane, and MeSH databases using the key search terms “treponema pallidum”, “sexually transmitted infections”, “pregnancy”, “congenital infection”, “syphilis”, and “congenital syphilis”, as well as their combinations. A total of 28 papers published over a ten-year period were included in the literature review. A clinical case was analyzed. **Results:** The impact of syphilis on pregnancy is quite evident. Our case showcased one of the most common impacts, i.e., premature birth, of congenital infections with associated bacterial meningitis, respiratory distress syndrome, multiple organ damage, and insufficient weight. Such associations with many adverse pregnancy outcomes as well as congenital syphilis and neonatal defects are often avoidable. **Conclusions:** Considering the potential consequences of infections, the issue of sexually transmitted diseases remains relevant, and improving diagnostic and treatment opportunities becomes of paramount importance as cases increase.

## 1. Introduction

Syphilis, a bacterial infection caused by Treponema pallidum spirochetes, affects a considerable number of expectant mothers in regions with high disease prevalence, leading to a notably elevated rate of a perinatal mortality and morbidity. The main clinical symptoms of syphilis occur after a 21-day incubation period. Painless anogenital or oral mucosal ulcers and regional lymphadenopathy are typical to the primary syphilis infection. About 25% of cases of an untreated primary syphilis progress to secondary syphilis within 6–8 weeks; this stage is characterized by a diffuse rash, generalized lymphadenopathy, and *condyloma lata*, which can lead to syphilitic chancres. The latent stage of the disease, which is further categorized into early (up to 12 months after infection) and late (from 12 months after infection) stage, is characterized by a positive serological test without manifesting clinical signs. The latent stage is followed by tertiary syphilis, which is depicted by inflammatory lesions in the cardiovascular, nervous, and musculoskeletal system, as well as skin, typically occurring 15–30 years after the onset of the untreated infection. It is mentionable that advanced stages of the disease may manifest as neurosyphilis [1].

Syphilis during pregnancy is the second-most common preventable cause of a late fetal death worldwide (second only to malaria) and is associated with low birth weight, neonatal infections, and preterm birth [2]. The infection caused by Treponema pallidum can be transmitted through sexual contact or vertically from mother to fetus in utero (less commonly, during childbirth through contact between the newborn and maternal tissues) in all stages of infection (primary, secondary, latent, or tertiary) [1,3]. Untreated syphilis is associated with a high likelihood of transplacental transmission and up to an 80% increased risk of adverse pregnancy outcomes: intrauterine fetal demise, anomalies of fetal development, preterm birth, stillbirth, low birth weight, congenital syphilis, or neonatal death [4,5,6]. Cases of fetal growth retardation are also described [1]. However, these adverse outcomes can be mitigated through timely antenatal screening and treatment. As stated in the WHO guidelines, a single 2.4 million I.U. of penicillin G injected intramuscularly is recommended for early syphilis treatment during pregnancy. Despite widespread screening programs in many countries, perinatal and congenital infections remain a significant public health matter.

## 2. Case Report

A 34-year-old pregnant woman at the 33rd week of gestation was administered to the Obstetrics Emergency Department due to sparse bloody vaginal discharge.

This was her third pregnancy. In the past, she had one miscarriage and one uncomplicated pregnancy and delivery. The newborn weight was 4500 g. At 10 weeks of her current gestation, the rapid plasma reagin (RPR) test was positive (1:16), and the patient was consulted by an infectious disease specialist. An additional Treponema pallidum Hemagglutination test (TPHA) was taken and came back positive. The patient was referred for a consultation by a dermatovenerologist; however, she did not make an appointment. According to the patient’s health records, she was treated by a dermatovenerologist for secondary syphilis infection a year prior the current pregnancy occurred. Two injections of Benzylpenicillin benzathine 2.4 million I.U. were given to the patient. According to the woman, her partner was treated as well. During this pregnancy, the patient was diagnosed with gestational diabetes (A1GDM). At 17 and 30 weeks of gestation, the patient was consulted at a Perinatology clinic. The urinary bladder of the fetus was subjectively larger than normal; thus, a following ultrasound screening was recommended at 36 weeks of gestation. No other fetal development abnormalities were suspected.

At the Obstetrics Emergency Department, a few irregular uterine contractions were observed. Few episodes of fetal bradycardia were noticed while conducting a nonstress test. Fetal movements were present. During a vaginal examination, a small amount of brown bloody discharge was visible. Transvaginal ultrasound revealed a 38 mm length cervix, with closed internal *os*. A transabdominal ultrasound examination of the fetus was performed, and the following pathological changes were first observed: polyhydramnios (AFI 307 mm) and suspected fetal macrosomia (fetus abdominal circumference > 97.9%). Fetoplacental circulation was evaluated using color flow Doppler and was considered normal, with the umbilical artery parameters listed as follows: *a. umbilicalis* S/D: 2.36; *a. umbilicalis* RI: 0.58; *a. umbilicalis* PI: 0.9. Placenta was attached to the anterior wall of the uterus with no signs of placental abruption.

The patient was admitted to the Pregnancy Pathology Department for constant maternal and fetal monitoring. Blood samples were taken. C-reactive protein was 16.7 mg/L. The patient was consulted by a dermatovenerologist, and it was recommended to perform RPR and TPHA tests, both of which came back positive. Four hours past the admission, the patient complained about uterine contractions, occurring every 4 min. A transvaginal sonography was carried out, the cervix length was 26 mm, it was dilated 7 mm; a heterogeneous mass, possibly a clot, was visible in the cervical canal. Intrauterine infection was suspected, and it was decided not to induce tocolysis. One dose of Betamethasone 12 mg was given intravenously to initiate the maturation of the fetal lungs.

Sixteen hours after the admission, a cardiotocography (CTG) became pathological; late decelerations and reduced variability were present. The uterus was rigid at palpation. A small amount of brown bloody discharge was again visible during a vaginal examination. Partial placental abruption was suspected, and it was decided to perform an urgent cesarean section.

**Figure 1:**
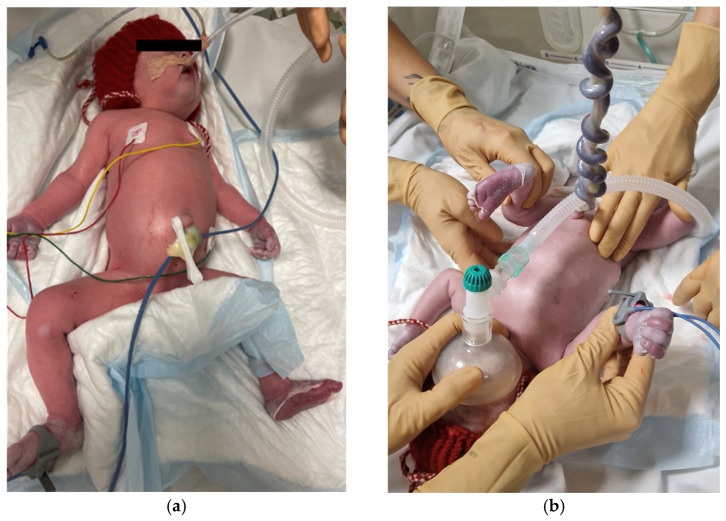
Condition of a newborn after the delivery. (**a**) Abdomen is enlarged due to hepatosplenomegaly. (**b**) Peeled skin of palms and soles is visible.

A premature female newborn, 2250 g in weight and 45 cm in length, was delivered. The umbilical cord blood pH was 7.30. The Apgar score was four after 1 min, six after 5 min, and seven after 10 min. The newborn was intubated and immediately transferred to a Neonatal Intensive Care Unit. Symptoms of severe respiratory failure and impaired microcirculation were observed. The skin of palms and soles was peeling (Figure 1). A petechial rash on the back and pale spots on the calves were noticeable. The abdomen was tight, and hepatosplenomegaly and ascites were detected. Two erythrocyte mass transfusions were given for the treatment of a severe anemia. Anemia, leukocytosis and elevated C-reactive protein (CRP) (74.0 mg/L), and Thrombocytopenia were present. Elevated liver enzymes were seen. Serological samples were taken to investigate if the syphilis infection was transferred from the mother to the newborn. TPHA (4+) and RPR (1:16) tests were both positive, and Treponema pallidum IgM were detected. A lumbar puncture was performed, and the cerebrospinal fluid (CSF) analysis showed elevated protein levels (6.670 g/L) and Treponema pallidum s/co of 12.53, indicating a treponemal infection. Based on clinical symptoms and laboratory test results, an early congenital syphilis infection was diagnosed. The neonate underwent a comprehensive examination to evaluate for possible multisystemic pathologies. Early cardiac ultrasound revealed right ventricular dilation and suboptimal left ventricular contraction (an ejection fraction of 57%). A grade III tricuspid valve insufficiency was identified, with a systolic gradient of 51 mmHg. Additionally, a significant bilateral patent ductus arteriosus was observed. Increased pulmonary vascular resistance, typical in neonates, was also noted. Further cardiac findings included grade II mitral regurgitation, grade I aortic regurgitation, and a secondary atrial septal defect. The abdominal ultrasound showed hepatosplenomegaly, while the cranial ultrasound indicated a slightly enlarged corpus callosum. Chest, abdominal, and long bone X-rays were performed, revealing signs consistent with neonatal respiratory distress syndrome, underdeveloped lungs, and a normal bone structure with no significant abdominal findings. The ophthalmological examination revealed no pathologies.

Respiratory failure did not progress and, eventually, reduced. Abdominal volume decreased. Antibiotic treatment was prescribed: penicillin was given intravenously for 21 days and gentamicin for five days. After delivery, parenteral nutrition was initiated, and later the newborn was fed with breast milk and a formula. Three weeks and five days after the birth, serological and tests were repeated, and the RPR titer was 1:8. The thrombocyte levels normalized. One month after birth, the newborn was discharged from the Neonatal Department in a stable condition.

After delivery, the mother was consulted by a dermatovenerologist for a second time. It was concluded that the patient needed an immediate antibiotic treatment and Benzylpenicillin benzathine therapy of 1 million I.U. intramuscularly (one dose every 3 h for 24 h period) was initiated. Subsequently, Benzylpenicillin benzathine 2.4 million I.U. was prescribed (one dose every 7 days for 3 weeks). It was also decided that the patient can breastfeed the newborn. The patient was also consulted by a neurologist. No neurological symptoms were present; however, it was decided that for the diagnostical and treatment purposes, a more detailed examination is necessary. A head computer tomography (CT) imaging was performed to exclude focal neurological damage, and no pathological findings were seen. To determine if neurosyphilis was present, a Venereal Disease Research Laboratory test from cerebrospinal fluid (VDRL-CSF) was performed; no pathological changes were observed. Additionally, an ophthalmological examination was conducted, and no significant changes in retinal fundus were seen.

**Figure 2:**
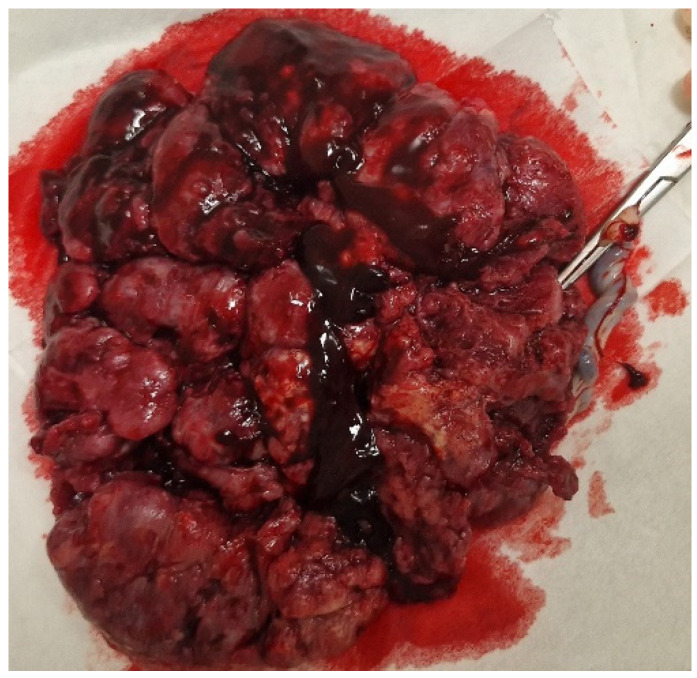
Macroscopic view of the placenta with visible hemorrhages.

The placenta and amniotic sac were sent for a histological examination. The macroscopic evaluation revealed the maternal part to be brown-colored with hemorrhages and a stiffening area (3 × 2 cm) in the center (Figure 2).

**Figure 3:**
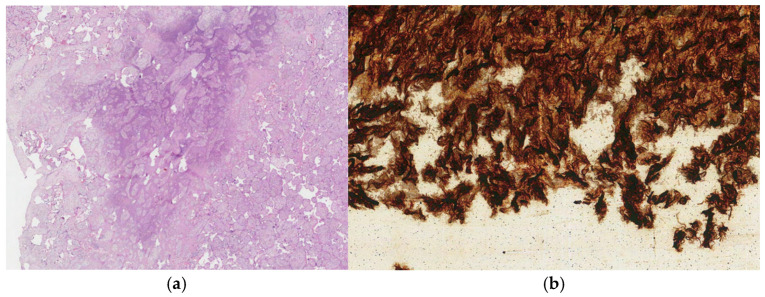
(**a**) Leukocyte infiltration in the placenta. The tissue was stained with Hematoxylin and Eosin. (**b**) Spirochetes detected in the umbilical cord. The tissue was stained with Warthin–Starry.

In the cross-section view, small ischemic foci were observed. The fetal part was with no visible changes. Microscopically, the necrosis of chorionic villi was confirmed. In the chorionic plate, umbilical cord, and amniotic sac, polymorphonuclear leukocyte infiltration was abundant. Spirochetes were detected in the umbilical cord stroma by the Warthin–Starry method (Figure 3).

## 3. Discussion

In the World Health Organization (WHO) Global Health Sector strategy on sexually transmitted infections for 2016–2021, one of the objectives was to eliminate congenital syphilis as a public health problem in 80% of countries (i.e., 50 cases of congenital syphilis per 100,000 live births in a country). Now, the 2022–2030 strategy aims to reduce the number of new syphilis cases by 90% by 2030 [7]. According to WHO recommendations for the antenatal screening and treatment of syphilis, all pregnant women should be tested for syphilis during their first antenatal visit and again in the third trimester [8]. Since individuals with syphilis are at a higher risk of acquiring other sexually transmitted diseases (STIs), a comprehensive diagnosis of other STIs is recommended. All individuals with syphilis should also be tested for human immunodeficiency virus (HIV) and hepatitis C virus (HCV) in the case of existing epidemiological risk factors in the region [9]. In our case, according to the national guidelines [10], the patient was supposed to be tested for syphilis in a first trimester of each pregnancy. However, we do not have any data of her previous pregnancies, and it is possible that early syphilis might have been missed. The rapid plasma reagin test during her first antenatal visit of the last pregnancy was positive; however, the patient did not initiate further investigations.

From the second week of gestation, *T. pallidum* can be transmitted to the fetus through the placenta, leading to miscarriage [4]. Thus, all pregnant women who have experienced miscarriage, stillbirth, or early neonatal death should be tested for syphilis. Subsequently, treatment for confirmed infection should be advised in order to protect the woman’s health and potential future pregnancies from related complications [4]. It is notable that since our patient had an early miscarriage, syphilis could have been the potential cause of the event. From the 16th week of gestation, the pathogen spreads to various fetal organs, causing microvascular proliferation and inflammation in both the placenta and the umbilical cord. Villitis, chorioamnionitis, and villous immaturity are considered the most common placental abnormalities caused by syphilis [11,12]. Both pathogenic conditions impair fetal development and can be a cause of preterm birth or intrauterine fetal death [4]. In our case, at 17 weeks of gestation, the pathology of fetal urinary tract was suspected. In addition, the histological examination of the placenta and fetal membranes after the delivery revealed signs of chorioamnionitis, chronic inflammation, and villi necrosis.

Syphilis-related adverse pregnancy outcomes are well known, although new insights on the possible role of the timing of the treatment, coinfections, and additional factors are being discussed. Miscarriage is often associated with syphilis. Studies show that maternal infection correlates with a 1.24 time increased risk of any type of miscarriage [13]. A miscarriage rate of 1.4% was observed in the population of syphilis-infected women [14]. It is noteworthy that a history of STIs (including syphilis) also significantly correlates with an increased risk of spontaneous miscarriage and medically induced miscarriage/delivery [15].

Preterm birth is a widely described adverse pregnancy outcome in the population of women with syphilis. According to the study of H. Liu and colleagues [14], delivery before 37 weeks of gestation was the most common adverse pregnancy outcome, significantly more frequent among untreated syphilis-infected women. Similar results were found in other studies conducted in China: compared to immediately treated patients, a significantly increased risk of preterm birth was found in pregnant women who started standard antibiotic therapy only in the third trimester or did not receive it at all [16]. Moreover, association between syphilis and preterm birth is particularly strong in the case of inadequate treatment—the risk is said to increase by up to 2 times [1]. A study by R. Gao et al. concluded that among the three STIs studied during pregnancy (syphilis, gonorrhea, and chlamydia), pregnant women with syphilis experienced preterm birth most frequently (13.3% of all cases) [17]. Notably, the coinfection of syphilis and other STIs further increases the likelihood of adverse outcomes compared to monoinfection [18].

Low newborn weight as a consequence of syphilis during pregnancy was described during a cross-sectional study conducted in Brazil, but nearly half of the subjects did not adhere to the treatment regimen or were not treated at all [16,19]. According to Z. Wan et al., low birth weight was significantly more common in women who were untreated or started the treatment in the third trimester as well [16].

Stillbirth risk, early neonatal death, and congenital defects are described less frequently. Late fetal death occurs significantly more often in untreated and inadequately treated patients and in pregnant women who started antibiotic therapy in the third trimester [16]. According to another study, syphilis during pregnancy is considered a significant predisposing factor for stillbirth—the risk increased by up to 3.4 times for infected women living in Brazil [13]. Congenital defects, severe neonatal infections, and cases of congenital syphilis may occur; however, statistically significant correlation with treatment status for these pathologies is not observed.

Gestational hypertensive disorders are also associated with *T. pallidum* infection. The study of Lindey R. Felske et al. conducted in the United States aimed to clarify the impact of syphilis, other STIs, and their coinfections on pregnancy. Data analysis revealed a higher risk of gestational hypertension in the study group, especially among syphilis-infected women [18].

It is too curious to consider the impact of concordance of maternal and partner infections on pregnancy—it is observed that partner coinfection increases the frequency of adverse pregnancy outcomes (a nearly 3 times higher risk of a late fetal death and a 1.3–1.5-times higher risk of a preterm birth and a low birth weight); nonetheless, adequate treatment can reduce such outcomes. It is worth mentioning that in cases of partner coinfection, pregnant women treated with two courses of penicillin G have a lower risk of complications during pregnancy compared to the ones given one course of treatment. With this regimen, the frequency of adverse outcomes among concordant and discordant couples remains similar. In addition, some studies conclude that in cases where a single-dose therapy is administered, women with infected partners experience complications more often during pregnancy [20].

Congenital infection occurs nearly four times more often in inadequately treated women, while antibiotics given in the first trimester prevent congenital syphilis entirely [16]. A. Lopez et al. emphasized that late maternal syphilis diagnosis and inadequate antimicrobial therapy before the delivery are associated with a high risk of the newborn being infected with congenital syphilis; thus, the timely diagnosis and standardized sequential treatment of the mother and partner are considered to be crucial [21]. However, many women still lack the resources to properly treat syphilis, even with an early diagnosis. The lack of perinatal care and timely screening opportunities hinders the prevention of congenital syphilis, especially in low- and middle-income countries with limited resources [1]. Speaking of diagnostic measures, the direct detection of spirochetes in a biological sample is important for the final diagnosis (dark-field microscopy being the gold standard); however, syphilis is usually diagnosed based on clinical symptoms and serological (both non-treponemal and treponemal) tests [9]. Treponemal-specific tests include TPHA and Enzyme-Linked Immunosorbent Assay (ELISA). The two main non-treponemal tests are VDRL and RPR, both based on the detection of patient serum IgM and IgG antibodies against cardiolipin, lecithin, and cholesterol [9]. These testing methods have lower sensitivity compared to treponemal tests; thus, early syphilis infection may not be detected [9]. It is noteworthy that the same antibodies can be found due to recent vaccination, fever, pregnancy, or chronic illnesses (intravenous drug use, systemic lupus erythematosus, and cancer), as well as aging processes. Therefore, false-positive results are also possible. Although seroconversion usually occurs around the third week of infection, the process can take up to six weeks after infection; hence, women with very early primary syphilis may initially receive falsely negative serological test results [22]. As stated before, the Centers for Disease Control and Prevention (CDC) and WHO recommend regular serological testing for pregnant women in high-prevalence infection areas during the first antenatal visit, at 28 weeks of gestation, and during labor, but recommendations vary geographically [23].

Many publications highlight the significance of appropriate antibacterial therapy in the context of pregnant women with syphilis. For instance, one course of penicillin prescribed before 28 weeks of pregnancy is particularly crucial in preventing adverse infection outcomes [14]. A significant increase in the risk of pregnancy complications was found for women who began standardized penicillin treatment late, only in the third trimester [16,24]. With respect to our patient, the second serological RPR blood test for syphilis diagnosis had to be performed at 28 weeks of gestation because of the increased risk of adverse pregnancy outcomes when treatment is initiated after this period. The initial RPR titre was 1:16. The status of her partner was unknown, and proper treatment was not ensured due to noncompliance.

The effective treatment of syphilis is easily achievable because the usual therapy is penicillin G, and bacterial resistance to this particular antibiotic is almost non-existent [23]. As stated in the WHO guidelines, a single 2.4 million I.U. of penicillin G (Benzylpenicillin) injected intramuscularly is recommended for early syphilis treatment during pregnancy. Second-line therapy is suggested as 600,000 I.U. of procaine penicillin intramuscularly daily for 10–14 days. The late syphilis and syphilis of unknown duration should be treated with a single 2.4 million I.U. of penicillin G intramuscularly once a week for three weeks. For individuals allergic to penicillin, doxycycline is prescribed orally for early syphilis treatment, 200 mg for 14 days; late-stage syphilis is treated with 200 mg of doxycycline orally for 21–28 days. Penicillin desensitization and treatment with Benzylpenicillin G are recommended for pregnant women allergic to penicillin [8,9]. Alternative treatment algorithms are known. For instance, in China, two-course penicillin G therapy (two courses of treatment, three injections per course, and one injection per week) is recommended. Nevertheless, it is claimed that different methods do not significantly differ in terms of syphilis outcomes [14,25]. It is worth mentioning that treatment with penicillin G remains the only effective method to prevent cases of congenital syphilis [23]. According to WHO assessment data from 2008 and 2012, appropriate syphilis treatment in pregnant women (at least one dose of 2.4 million I.U. of penicillin G) possibly reduced the number of early or late fetal deaths, preterm births, low birth weight infants, newborn illnesses, and deaths by 82%, 65%, 80%, and 97%, respectively [5]. Point-of-care treponemal tests performed during antenatal care visits increase diagnostic and treatment rates and are associated with higher numbers of healthy pregnancies in regions lacking standardized diagnostic strategies, laboratory testing capabilities, and non-treponemal syphilis tests [26]. With that in mind, there is a steadily growing need for such testing and treatment methods, where key tests, results, and treatment are provided during a preventive visit, and the patient is informed immediately. Such a strategy could have potentially prevented the adverse outcome in our case. Currently, many studies on economic viability suggest that managing the situation through point-of-care methods is more financially effective compared to laboratory testing and syndromic management methods or when there are no preventive programs [27]. It is also worth discussing that universal screening might help to reduce stigma and discrimination related to STIs and promote public engagement [28].

### Patient Noncompliance and Late Intervention: A Case-Based Perspective

A thorough anamnesis, especially regarding previous pregnancies, is essential to fully understand the current clinical situation. Another aspect that should be taken into consideration is that ensuring patient compliance can be a challenge. Here, compliance could have potentially led to a more favorable clinical outcome. As noted earlier, the late diagnosis resulting from neglected prenatal care, the potentially insufficient investigation into the causes of previous miscarriage, and the possible untreated infection of the partner, along with delayed treatment, are believed to have negatively impacted the course of the disease and neonatal state. Therefore, in difficult cases with potentially socially vulnerable patients, as our patient was, the use of point-of-care treponemal tests during antenatal visits, along with accessible point-of-care treatment options, could help to avoid adverse pregnancy outcomes.

## 4. Conclusions

Syphilis is still considered the second-most common preventable cause of a fetal loss in a late pregnancy. Despite widespread screening programs in many countries, perinatal and congenital infections remain a significant public health issue. The impact of syphilis on pregnancy is quite evident. Our case showcased one of the most common impacts, i.e., premature birth, of congenital infections with associated bacterial meningitis, respiratory distress syndrome, multiple organ damage, and insufficient weight. Such associations with many adverse pregnancy outcomes as well as congenital syphilis and neonatal defects are often avoidable. The importance of the timely initiation and continuity of treatment is of major importance—the 28th gestational week is considered a critical threshold beyond which the risk of adverse pregnancy outcomes and harm to the fetus significantly increases, regardless of antibiotic therapy. The frequency of complications may also increase in cases of partner coinfection. To prevent the burden of complications throughout life, timely diagnosis and treatment are encouraged, which could be facilitated through prevention programs and stigma reduction, especially among socially vulnerable and high-risk groups. A responsible attitude of a patient towards pregnancy as well as compliance with the recommendations of specialists is also of crucial importance. Although syphilis screening is widely known and developed in many countries, low-income nations often lack opportunities for recommended diagnostics, particularly vital for pregnant individuals. Considering the potential consequences of infections, the issue of sexually transmitted diseases remains relevant, and improving diagnostic and treatment opportunities becomes of paramount importance as cases increase.

## Data Availability

The original contributions presented in this study are included in the article. Further inquiries can be directed to the corresponding authors.

## Abbreviations

RPR	Rapid Plasma Reagin
TPHA	Treponema Pallidum Hemagglutination Test
AFI	Amniotic Fluid Index
CTG	Cardiotocography
CRP	C-Reactive Protein
CSF	Cerebrospinal Fluid
CT	Computer Tomography
VDRL-CSF	Venereal Disease Research Laboratory Test from Cerebrospinal Fluid
WHO	World Health Organization
STIs	Sexually Transmitted Diseases
HIV	Human Immunodeficiency Virus
HCV	Hepatitis C virus
ELISA	Enzyme-Linked Immunosorbent Assay
CDC	Centers for Disease Control and Prevention

## References

[1] Torres R.G., Mendonça A.L.N., Montes G.C., Manzan J.J., Ribeiro J.U., Paschoini M.C. (2019). Syphilis in Pregnancy: The Reality in a Public Hospital. Rev. Bras. Ginecol. Obs..

[2] WHO Publishes New Estimates on Congenital Syphilis. https://www.who.int/news/item/26-02-2019-who-publishes-new-estimates-on-congenital-syphilis.

[3] Schlueter A., Doshi U., Garg B., Hersh A.R., Caughey A.B. (2022). Adverse pregnancy outcomes associated with maternal syphilis infection. J. Matern. Fetal Neonatal Med..

[4] Zhang X., Yu Y., Yang H., Xu H., Vermund S.H., Liu K. (2018). Surveillance of Maternal Syphilis in China: Pregnancy Outcomes and Determinants of Congenital Syphilis. Med. Sci. Monit. Int. Med. J. Exp. Clin. Res..

[5] Korenromp E.L., Rowley J., Alonso M., Mello M.B., Wijesooriya N.S., Mahiané S.G., Ishikawa N., Le L.V., Newman-Owiredu M., Nagelkerke N. (2019). Global burden of maternal and congenital syphilis and associated adverse birth outcomes-Estimates for 2016 and progress since 2012. PLoS ONE.

[6] Syphilis—Global. https://www.who.int/health-topics/syphilis.

[7] Global Health Sector Strategies. https://www.who.int/teams/global-hiv-hepatitis-and-stis-programmes/strategies/global-health-sector-strategies.

[8] World Health Organization (2017). WHO Guideline on Syphilis Screening and Treatment for Pregnant Women.

[9] Janier M., Unemo M., Dupin N., Tiplica G.S., Potočnik M., Patel R. (2021). 2020 European guideline on the management of syphilis. J. Eur. Acad. Dermatol. Venereol..

[10] Akušerinė Metodika_Antenatalinė Priežiūra_SAM_2019 07 13.pdf. https://sam.lrv.lt/uploads/sam/documents/files/Akus%CC%8Cerine%CC%87%20metodika_Antenataline%CC%87%20priez%CC%8Ciu%CC%84ra_SAM_2019%2007%2013.pdf.

[11] Marais Y.A., Mason D., Barnard A., Saaiman C.R., Els H.C., Kluge J., Glass A.J., Wright C.A., Schubert P.T. (2023). Placental Syphilis: A Comprehensive Review of Routine Histomorphology, HIV Co-infection, Penicillin Treatment, Immunohistochemistry, and Polymerase Chain Reaction. Fetal Pediatr. Pathol..

[12] Kim C.J., Romero R., Chaemsaithong P., Kim J.S. (2015). Chronic Inflammation of the Placenta: Definition, Classification, Pathogenesis, and Clinical Significance. Am. J. Obstet. Gynecol..

[13] Yang L., Cambou M.C., Segura E.R., De Melo M.G., Santos B.R., Dos Santos Varella I.R., Nielsen-Saines K. (2022). Patterns of pregnancy loss among women living with and without HIV in Brazil, 2008–2018. AJOG Glob. Rep..

[14] Liu H., Chen N., Yu J., Tang W., He J., Xiao H., Lin S., Hu F., Feng Q., Tucker J.D. (2019). Syphilis-attributable adverse pregnancy outcomes in China: A retrospective cohort analysis of 1187 pregnant women with different syphilis treatment. BMC Infect. Dis..

[15] Zeng M., Yang L., Mao Y., He Y., Li M., Liu J., Zhu Q., Chen L., Zhou W. (2022). Preconception reproductive tract infections status and adverse pregnancy outcomes: A population-based retrospective cohort study. BMC Pregnancy Childbirth.

[16] Wan Z., Zhang H., Xu H., Hu Y., Tan C., Tao Y. (2020). Maternal syphilis treatment and pregnancy outcomes: A retrospective study in Jiangxi Province, China. BMC Pregnancy Childbirth.

[17] Gao R., Liu B., Yang W., Wu Y., Wang B., Santillan M.K., Ryckman K., Santillan D.A., Bao W. (2021). Association of Maternal Sexually Transmitted Infections With Risk of Preterm Birth in the United States. JAMA Netw. Open.

[18] Felske L.R., Castillo E., Kuret V., Metcalfe A. (2022). Comparing adverse neonatal and maternal outcomes of chlamydia, gonorrhoea, and syphilis infections and co-infections in pregnancy. Paediatr. Perinat. Epidemiol..

[19] Padovani C., Oliveira RR de Pelloso S.M. (2018). Syphilis in during pregnancy: Association of maternal and perinatal characteristics in a region of southern Brazil. Rev. Lat. Am. Enferm..

[20] Zhang X.H., Chen Y.M., Sun Y., Qiu L.Q., Chen D.Q. (2019). Differences in maternal characteristics and pregnancy outcomes between syphilitic women with and without partner coinfection. BMC Pregnancy Childbirth.

[21] Lopez A., Lee S.J., Bullard J. (2022). Syphilis in pregnancy and infant outcomes in Manitoba. Paediatr. Child Health.

[22] Hoffman B.L., Schorge J.O., Halvorson L.M., Hamid C.A., Corton M.M., Schaffer J.I. (2020). Gynecologic Infection. Williams Gynecology.

[23] Van Gerwen O.T., Muzny C.A., Marrazzo J.M. (2022). Sexually transmitted infections and female reproductive health. Nat. Microbiol..

[24] Li Z., Wang Q., Qiao Y., Wang X., Jin X., Wang A. (2021). Incidence and associated predictors of adverse pregnancy outcomes of maternal syphilis in China, 2016–2019: A Cox regression analysis. BJOG Int. J. Obstet. Gynaecol..

[25] Hu F., Guo S.-J., Lu J.-J., Zhu S., Hua N.-X., Song Y.-Y., Liang J.-J., Yu J., Lin S.-F. (2020). The Effect of Different Treatment Regimens and Multiple Risk Factors on Adverse Pregnancy Outcomes among Syphilis-Seropositive Women in Guangzhou: A Retrospective Cohort Study. BioMed Res. Int..

[26] Brandenburger D., Ambrosino E. (2021). The impact of antenatal syphilis point of care testing on pregnancy outcomes: A systematic review. PLoS ONE.

[27] Saweri O.P.M., Batura N., Al Adawiyah R., Causer L.M., Pomat W.S., Vallely A.J., Wiseman V. (2021). Economic evaluation of point-of-care testing and treatment for sexually transmitted and genital infections in pregnancy in low- and middle-income countries: A systematic review. PLoS ONE.

[28] (2011). Screening for Chlamydia and Gonorrhea During Pregnancy: A Health Technology Assessment. CADTH Report/Project in Briefs.

